# Potential Synergistic Roles of Ketogenic Diet and Stem Cell Therapy in Parkinson's Disease: A Narrative Review

**DOI:** 10.7759/cureus.103304

**Published:** 2026-02-09

**Authors:** Princy Sindurakar, Vasavi R Gorantla, Dovenia Ponnoth

**Affiliations:** 1 Biomedical Sciences, West Virginia School of Osteopathic Medicine, Lewisburg, USA; 2 Medical Education, California University of Science and Medicine, Colton, USA; 3 Research, West Virginia School of Osteopathic Medicine, Lewisburg, USA

**Keywords:** ketogenic diet therapy, levodopa, neurodegeneration, parkinson's disease, stem cell therapy

## Abstract

Parkinson's disease (PD) is a complex neurodegenerative disorder and is the second most common, after Alzheimer's disease. Marked by the gradual loss of dopaminergic signaling and accumulation of abnormal protein aggregates, this condition presents in patients with a combination of motor and non-motor symptoms. While first-line treatments often include exogenous dopamine therapy, such as levodopa-carbidopa, and surgical procedures or deep brain stimulation, these mainstream therapeutics often fail to improve disabling symptoms of PD, induce serious side effects, and have no effect in controlling disease progression. An alternative approach of cell-based therapies has shown to be an effective therapeutic in reducing neurodegeneration. Additionally, diet and exercise modifications have played crucial roles in the management of the condition. Given that certain dietary regimens may be associated with stem cell renewal and regeneration, specifically in the context of tumors and cancer, a potential combination of a ketogenic diet, a high-fat and low-carbohydrate strict dietary regimen, and stem cell therapy with dopaminergic neurons could serve as a beneficial therapeutic option for PD patients, altering the underlying neurodegeneration. Based on several processes of PD pathophysiology, mainly mitochondrial dysfunction, defective protein clearance mechanisms, and neuroinflammation, treating patients with current therapeutics and implementing a ketogenic diet alongside stem cell therapy may significantly improve PD symptoms and progression. This narrative review was conducted using a structured literature search from PubMed and Google Scholar using terms such as PD, stem cell therapy, ketogenic diet, mitochondrial dysfunction, and neuroinflammation. Given the central roles of mitochondrial dysfunction, impaired protein clearance, and neuroinflammation in PD pathophysiology, the combined use of metabolic interventions and stem cell-based strategies represents an avenue for future investigation. Currently, there are no clinical trials using human models to establish the safety, feasibility, or efficacy of the combined therapy using the ketogenic diet and stem cell therapy in PD. Rigorous preclinical validation and well-designed clinical trials are required before such strategies can be considered viable disease-modifying options for PD.

## Introduction and background

Parkinson's disease (PD) is a complex neurodegenerative disorder that entails slowing of movements and at least one other symptom of tremor or rigidity. Often, there is a combination of non-motor and motor manifestations, with typical clinical features including resting tremors, bradykinesia, and rigidity [1].

Pathophysiological basis for metabolic therapies in PD

PD mainly stems from the loss of dopaminergic neurons in the substantia nigra (SN) and presents with Lewy bodies (LB), which are composed of abnormal deposits of protein known as alpha-synuclein in the brain and are associated with the progression of PD dementia [2]. While the cause of PD remains unknown, a number of genetic risk factors have been characterized, as well as several genes identified, which cause rare familial forms of PD [1]. Additionally, numerous environmental influences, such as smoking, caffeine consumption, vigorous exercise, and pesticide exposure, have been identified as risk factors of PD development [3].

Numerous processes have been implicated in PD. Amongst the different brain regions, the SN is one of the key regions of the midbrain that plays a crucial role in the regulation of the body's motor function, and the degeneration of dopaminergic neurons in the SN is a hallmark feature of PD [4]. This degeneration of neurons often results in reduced dopamine (DA) levels in the striatum, specifically in the caudal brain and nucleus [5]. A distinct feature of PD has been observed in transverse sections of the brainstem, where there is a significant loss of pigmentation in areas such as SN pars compacta (SNpc) and locus coeruleus, reflecting the death of dopaminergic neuromelanin-containing neurons and noradrenergic neurons, respectively. It is noted that studies found that postmortem PD brains show approximately 30% loss of dopaminergic neurons in SNpc by motor onset [6]. Following the appearance of motor symptoms, neuronal loss increases up to 60% or higher, which strongly correlates with the progressive severity of motor features and disease duration [7]. As a result of the neuronal loss, there are diminished DA levels in the striatum, which lead to cardinal motor symptoms in PD [1]. Additionally, there is a widespread cell loss in several subcortical nuclei, such as raphe nuclei, dorsal motor nucleus of the vagus nerve, hypothalamus, and subregions of SN, amongst others [8], which often mirrors PD disease duration and symptoms. Researchers investigated the extent of nigrostriatal degeneration in patients with PD at different disease durations from the time of diagnosis, finding that there was a modest loss of dopaminergic markers in the dorsal putamen following one year after diagnosis, while there was moderate to marked loss after three years and virtually complete loss of staining at four years after diagnosis. There was also a 50-90% loss of tyrosine hydroxylase-positive neurons from early time points and additional loss after, emphasizing the importance of developing biomarkers that allow PD diagnosis at early time points to ensure greater preservation of dopaminergic markers and higher neuroprotective effects [8].

As indicated earlier, one of the hallmarks of PD is the presence of LB, which are immunoreactive for protein alpha-synuclein, and Lewy neurites [9]. LBs consist of vesicular membrane structures and dysmorphic organelles with protein aggregates consisting of alpha-synuclein as the main component [10]. The two types of LB include classical brainstem and cortical LBs, with the two being morphologically distinct. There are also structures that resemble cortical LBs called pale bodies, considered to be precursors to the LBs. LB's primary structural component is filamentous alpha-synuclein, established to contribute to the abnormal pathology in PD, specifically from being abnormally phosphorylated and aggregated [1]. The aggregated alpha-synuclein serves as a major constituent of LB fibrils, a histopathological hallmark of PD, and may even be cytotoxic, exacerbating neuronal injury, disrupting mitochondrial function, and impairing autophagic clearance pathways [9,10]. In addition to alpha-synuclein, the molecular components of an LB include a number of proteins, such as ubiquitin, tau, parkin, heat shock proteins (HSP), and cytoskeletal proteins, proteasomal and lysosomal elements [11]. PD pathology was initially classified using a staging system based on the semiquantitative assessment of LB distribution, at postmortem, in a large autopsy series [12]. Each stage parallels the severity of symptoms in patients, ultimately with later stages affecting higher-order and functioning areas such as the prefrontal cortex and primary and motor sensory areas, which are directly involved in severe PD symptoms of gait problems and dementia. However, there have been subsequent studies that have criticized the Braak system for being based on the distribution of Lewy-related pathology rather than neuronal loss [1].

Another key element in the pathogenesis of both idiopathic and familial PD is mitochondrial dysfunction [13]. The dysfunction has been reported in various non-dopaminergic cells and tissue samples from human patients, transgenic mouse models, and fruit fly models of PD [14]. Early postmortem studies examining the SN of PD brains reported a deficiency of the mitochondrial complex I, which is a vital component of the electron transport chain [15]. Similarly, the abuse of the substance, 1-methyl-4-phenyl-1,2,3,6-tetrahydropyridine (MPTP), also causes permanent Parkinsonian symptoms, with postmortem examination revealing dopaminergic cell loss [1]. Many studies consist of mouse models using MPTP to study the pathophysiology of PD, as exposure to MPTP occurs through many known chemicals, such as insecticide rotenone or herbicides, environmentally [16]. MPTP, being a highly lipid-soluble organic compound, can easily cross the blood-brain barrier (BBB) and convert to its toxic metabolites, such as the MPP+ ion, if directly infused into the brain [16]. Studies have shown that MPTP, when oxidized, is taken up by DA neurons and leads to complex-1 inhibition [17]. Defects in this complex may be crucial in driving DA cell death due to energy depletion. Furthermore, alpha-synuclein is known to interfere with mitochondrial function as it can interact with the mitochondrial membrane and accumulate inside the organelles. This leads to damage to the complex activity, thus resulting in mitochondrial dysfunction and increased oxidative stress. The mitochondrial dysfunction can then impact protein degradation, as the protein aggregates can repair lysosomal function and mutant alpha-synuclein often reduces its own degradation, causing accumulation and localization within mitochondrial membranes [18]. Loss-of-function mutations in certain genes, such as the PTEN-induced putative kinase 1 (PINK1) and parkin gene, which are vital components of the pathways that regulate the removal of dysfunctional mitochondria, lead to impaired mitochondrial quality control and cause autosomal recessive PD [1]. Since neurons have a complex network of mitochondria ranging from dendrites to synaptic terminals, the role of mitochondria is essential for generating ATP, calcium buffering, and epigenetic signaling [19]. The mitochondrial theory of pathogenesis states that neurons naturally have high bioenergetic demand and rely heavily on mitochondria for ATP production, specifically increasing their bioenergetic needs, unlike many other cell types [13].

Mitochondria, thus, play a crucial role in the metabolic signaling and are a critical source of citrate, significantly impacting the production of acetyl-CoA and acetylation of proteins and DNA [13]. When mitochondrial function is compromised, SNc dopaminergic neurons are at the greatest risk for the development of PD since these neurons are constantly active and have extensive axonal arbors for transmitter release, requiring a high bioenergetic demand [20]. In the setting of mitochondrial dysfunction, neurons increasingly rely on plasma membrane L-type calcium channels, crucial for sustained release of DA [21], to drive mitochondrial oxidative phosphorylation. This compensatory mechanism is needed to meet the high bioenergetic needs of these neurons and maintain sufficient ATP production. However, persistent calcium influx during mitochondrial DNA deletions and impaired respiratory capacity can also promote excess production of damaging reactive oxygen species (ROS) and elevate basal mitochondrial oxidant burden [21]. This leads to a compromise in cellular function and has been linked to several genetic mutations seen in familial cases of PD involving mitochondrial quality control and lysosomal function [22].

Rationale for the KD in PD

Over the last few decades, there has been a variety of therapeutic developments for PD, yet there remains currently no cure for PD. With the prevalence of PD rising, the management and prevention of the condition have become crucial. Currently, PD symptoms are mainly managed by dopaminergic stimulation with new formulations of drugs and targeting pre-symptomatic disease stages [23]. We examined the literature to look at other alternative approaches to treating and managing PD. Multiple studies have found that the nutritional status of an individual with PD strongly relates to their quality of life through measures of one's body mass index, body composition, energy intake, and weight changes [24]. It has been speculated that, given nutrition and diet remain modifiable risk factors for chronic illnesses, they can potentially reduce PD risk and slow its progression. In neurodegenerative conditions, such as PD and Alzheimer's disease, there has been evidence of metabolic changes impacting glucose uptake, thus influencing symptoms from worsening cognitive and motor function. For instance, there is a known defect in the respiratory chain complex I activity, which impacts mitochondrial oxidative phosphorylation in the brain, particularly in PD pathology [24]. Certain dietary changes or implementations have shown improvement in neurodegenerative disorders such as PD and AD [25]. One of these dietary regiments include a KD, high-fat, moderate protein, and low-carbohydrate diet.

Stem cell therapy in PD: Evidence and challenges

Another treatment option for a spectrum of neurodegenerative disorders, including PD, has involved regenerative stem cell therapy [1]. Given the main hallmarks of PD pathogenesis, both KD and stem cell therapy have already shown to effectively reduce motor symptoms, independently. It has also been found that certain dietary interventions can significantly impact adult stem cells, particularly depending on the activity of ketone bodies, acting as a significant implication for regenerative medicine [25]. A combination of a KD and stem cell therapy can potentially serve great benefits to PD patients, targeting the management of PD.

Energy availability can profoundly impact stem cell function, specifically in muscle and intestinal stem cells (ISCs) [26]. Dietary regimens that implement caloric restriction, such as fasting and KD, exert notable effects on stem cells, improving health, delaying tissue aging, and elongating survival in flies, worms, and even mice models [27]. Most stem cells take up the circulating ketone bodies from the systemic environment, specifically acetoacetate. Ketone bodies can also influence the acetylation of soluble proteins, particularly the protein p53 [28], a key component of genetic stability, contributing to maintaining stem cell quiescence and preventing stem cell exhaustion.

Theoretical basis for combined KD and stem cell therapy

A study by Lu et al. studied how oxidative stress impacts stem cell function on many complex levels, using rat models, inducing rats with a spinal cord injury, and placing the group on a KD. Results showed that rats on a KD have high levels of nuclear factor erythroid-2-related factor 2, NRF2, a transcriptional factor activated by increased stress levels to stimulate transcription of antioxidant proteins [29]. KD suppressed the oxidative stress and inflammation along with the activation of NRF2 and nuclear factor-kB (NF-kB), signaling pathway in these rats with spinal cord injury. This thus regulates the self-renewal and regenerative capacity in various adult stem cell types, such as ISCs, neural stem cells (NSCs), hematopoietic stem cells (HSCs), and mesenchymal stem cells (MSCs). Increased NFR2 expression tends to concur with acetoacetate exposure. While outcomes of a KD can be variable, this intercalated relationship between ketone bodies and stem cell functioning suggests a potential addition to PD therapy [25].

Risks and translational barriers

This work is a narrative review and is therefore limited by its qualitative synthesis of the existing literature rather than a systematic or meta-analytic approach. As a result, its conclusions depend on the scope, quality, and potential biases of available studies, many of which are derived from preclinical models or small, heterogeneous clinical cohorts. Although both the KD and stem cell therapies show promise, animal and in vitro models do not fully recapitulate the complexity and heterogeneity of human PD. For KD, long-term adherence, tolerability, nutritional risks, and comorbidities in older PD populations remain significant clinical challenges. In addition, the lack of standardized dietary protocols and limited long-term clinical trial data restricts the strength of current recommendations. Stem cell therapies face substantial translational barriers, including issues of cell sourcing, graft survival, immune rejection, tumorigenicity, and long-term functional integration. The proposed combination of KD and stem cell therapy, while biologically plausible, currently lacks direct clinical evidence in PD populations. Future progress will require well-powered clinical trials, standardized outcome measures, and long-term safety and mechanistic studies.

## Review

Methods

A non-systematic search of the published literature was conducted across multiple databases and repositories, including Google Scholar, PubMed/MEDLINE (National Library of Medicine/NIH), PubMed Central (PMC), and other relevant biomedical indexing sources. Search terms and keyword combinations included “Parkinson’s disease”, “mitochondrial dysfunction”, “ketogenic diet”, “ketone bodies”, “metabolic therapy”, “oxidative stress”, “neuroinflammation”, “stem cell therapy”, “dopaminergic neurons”, “cell replacement”, and “regenerative medicine”, with additional citation chaining from reference lists of key articles to identify foundational and recent studies. Evidence was drawn from preclinical studies, specifically, in vitro and animal models, clinical studies, and review studies to provide the mechanistic context and summarize emerging therapeutic data. Studies were prioritized based on relevance to the review aims, the mechanistic or clinical interpretability of findings, and the degree to which they informed the proposed rationale for KD and stem cell therapies in PD. Given this is a narrative review, formal systematic review procedures (e.g., predefined eligibility criteria, Preferred Reporting Items for Systematic Reviews and Meta-Analyses (PRISMA) flow diagram, risk-of-bias scoring, or meta-analysis) were not performed. Findings were synthesized thematically to align with the manuscript subsections addressing PD pathophysiology, KD rationale and evidence, stem cell therapy evidence and challenges, and translational considerations for combined approaches.

Classical KD

With obesity being a major worldwide health hazard, the implementation of varied diet regimens was thought to mitigate the obesity epidemic to some extent. It is also noted that the number of Americans suffering from obesity, diabetes, and metabolic syndrome has been increasing [30]. A low-carbohydrate and high-fat KD emerged as a highly effective approach for rapid weight loss, enhancing mental clarity and increasing energy levels [31]. The KD has a macronutrient distribution typically ranging from approximately 55-60% fat, 30-35% protein, and 5-10% carbohydrate [32]. By significantly reducing carbohydrate consumption and increasing fat and protein intake, this diet, as mentioned, induces ketosis, a metabolic state where the body utilizes fat as a primary fuel source, not carbohydrates. A KD essentially restricts carbohydrate intake, thus decreasing insulin secretion and putting the body into a catabolic state, involving gluconeogenesis and ketogenesis. When glucose availability drops, the metabolic pathway switches to ketogenesis to provide an alternate energy source in the form of ketone bodies, which replace glucose as a primary source of energy. Low insulin secretion also leads to a decrease in stimulation of fat and glucose storage, resulting in increased fatty acids. These can be metabolized to primary ketone bodies, which accumulate in the body as the KD is sustained and serve as an alternative energy source for the body, establishing nutritional ketosis [33].

The use of diet as a treatment modality began with epilepsy; subsequently, intermittent fasting, fasting, and other dietary regimens started gaining momentum in this field as promising results were noted, which eventually led to the development and use of the KD to manage epilepsy [29]. The KD consists of a high-fat, moderate protein, and low-carbohydrate diet, as noted earlier, initially developed to control seizures as the treatment of epileptic children [34]. KD has been continued and used as an alternative to antiepileptic drugs for the treatment of refractory epilepsy and tends to be reserved for young patients with difficult epileptic conditions. KD has been documented as a treatment options of metabolic enzyme deficiencies, infantile spasms, Rett syndrome, tuberous sclerosis complex, and many others [35]. The goal of a KD is to induce a state of ketosis through increased production of ketone bodies, such as beta-hydroxybutyrate, acetoacetate, and acetone [36]. Ketone body metabolism is crucial in physiological homeostasis, with ketone bodies being produced mainly in the liver from acetyl-CoA derived from metabolic processes such as beta-oxidation [37]. Ketone bodies act as an alternative fuel source, mainly for the brain when the body undergoes nutrient deprivation. Prolonged caloric restriction and fasting reduce inflammation by the immune system, adapting to a low glucose state, utilizing processes such as mitochondrial fatty acid oxidation, ketogenesis, and ketolysis [38].

Using ketone bodies as the main energy source may provide clinical benefit for patients with neurodegenerative disorders [39]. A study in Japan examined the association between dietary glycemic index, glycemic load, and other carbohydrate variables and PD using data from a case-control study. Researchers found that dietary glycemic index was inversely associated with the risk of PD. While there is limited information about how to delay the onset or slow progression of PD, researchers have been attempting to understand dietary components involved in PD. Oxidative stress is a key link between PD and diet as most ROS in the cell are generated through mitochondrial metabolism of dietary macronutrients. Using rat models, researchers found that high glucose conditions relate to dynamic changes in mitochondrial morphology and overproduction of ROS [40]. Mimicking untreated diabetic conditions through extended exposure to increased glucose, the rat models were found to have a prolonged increase in ROS production with mitochondrial morphology changes within the rat livers. PD patients often have a deficiency in mitochondrial complex I activity and reduced mitochondrial DNA levels in the frontal cortex, associated with dementia and its development [41]. This is closely associated with increased superoxide radical, a primary ROS, production, which contributes to the death of dopaminergic neurons as mitochondrial complex I remains one of the primary sources of ROS [42]. In the MPTP mice model of PD, ketone bodies have been shown to exert neuroprotective activity within the brain by promoting antioxidant activity [43]. KD increases both glucose metabolism and ATP production in postnatal day 35 male rats [44]. These improvements have been attributed to ketone bodies acting as an alternative fuel, ultimately bypassing glycolysis and providing acetyl-CoA to enter the TCA cycle and facilitating ATP production. In a study exploring KD as an epileptic treatment, elevated medium chain fatty acids and the medium chain fatty acid, decanoic acid, led to marked increase in mitochondrial enzymes, complex I activity and catalase activity, thus an improvement in mitochondrial function and mitochondrial biogenesis [45]. KD increases mitochondrial mass and functional competence via peroxisome proliferator-activated receptor gamma-coactivator-1 alpha, which regulates mitochondrial biogenesis, mitochondrial sirtuins, and the uncoupling protein, UCP2 [46].

The main machinery of mitochondrial dynamics consists of three GTPases that fuse and divide the mitochondrial membranes: mitofusin 1 and 2 (mfn1 and mfn2), opa1, and dynamin-related protein 1 (drp1), which are involved in membrane fusion. Several studies have found that there was a reduction in mitochondrial number, disruption of mitochondrial membranes in muscle tissues of PD patients, abnormal mitochondrial distribution, variation in mitochondrial size, and swelling in biopsies taken from PD patients. An overexpression of a mutant form of alpha-synuclein in mice also causes abnormalities in mitochondrial structure and function, leading to progressive neuronal death through lysosomal dysfunction and alteration of calcium homeostasis in PD and other neurodegenerative disorders [47]. A high-fat diet (HFD) rich in saturated acids has facilitated mitochondrial fission by elevated levels of drp1 and downregulation of mfn2 with specific effects of each type of saturated fatty acids [48]. For instance, omega-3 polyunsaturated fatty acids have shown to improve mitochondrial function by reducing reactive species production and promoting mitochondrial fusion, increasing levels of mfn2 and ATP levels [46].

In addition to a tetrad of motor deficits, PD involves disorders in multiple systems with prominent neuroinflammation and immune dysfunction, leading to non-motor symptoms [49]. KD has been increasingly known for its broad efficacy in many experimental models, such as animal models with prominent inflammatory changes in the brain [50]. As mentioned, the classic KD is composed of a 4:1 ratio of fats to proteins and carbohydrates, reducing glucose utilization. In order to control conditions such as epilepsy, KD influences neuronal excitability, specifically with ketone bodies inhibiting the release of glutamate by competing with chloride, which directly activates VGLUT2, a glutamate transporter, at the site of allosteric regulation [51]. Similarly, neuroinflammation plays a role in many neurological disorders, including epilepsy. It has also been hypothesized that neuroinflammatory mechanisms might be potential causes of neuronal loss in PD [52]. Following cellular responses to neurodegeneration, mediated by activated glial and peripheral immune cells, certain events, such as oxidative stress and cytokine-receptor mediated apoptosis, contribute to dopaminergic cell death, thus PD development and progression. One study characterizing and comparing extended peripheral T-lymphocyte populations between PD patients versus normal subjects resulted in patients with PD having significantly decreased T cell ratios and increased ratios of interferon-gamma producing and interleukin-4 producing T cells [53]. PD patients do have increased levels of cytokines and growth factors, which can suggest that they may be produced as compensatory responses in the nigrostriatal dopaminergic regions in PD [54].

In mouse models, researchers used a paradigm of prolonged breastfeeding by weaning mice to a KD diet or to a regular standard diet, finding that brain growth and weight were comparable in both groups. The study investigated whether brain metabolism was attuned to a nutritional paradigm by analyzing key metabolic enzymes and transporters in the cortex of the two mouse groups. Data showed that a KD was validated in a long-term feeding paradigm, which induced chronic ketosis in the brain without causing brain damage. The cortical cell types consequently followed specific strategies to adapt their metabolism to ketosis, particularly showing that KD strongly modified the proteome profile of astrocytes, neurons, and microglia compared to standard-diet control mice. Additionally, in tissues of KD-fed mice, endothelial cells increased expression of metabolic transporters, but not that of ketolytic enzymes. KD increased both neuronal oxidative phosphorylation and other major energy metabolic pathways. Although strict dietary regimens come with the issue of a lack of compliance from patients, KD has strongly shown to rebalance CNS and peripheral metabolism in inflammatory diseases, particularly encephalopathies. Raising steady-state levels of ketone bodies, specifically their import to the CNS and usage, could have implications for the management of many other neurodegenerative diseases [55].

Stem Cell Therapy

Additionally, stem cell therapies are one of the most promising approaches to regenerative treatment that could be used in many patients with PD. Currently, stem cell therapies have continued to be studied and utilized for a variety of different illnesses, such as ocular diseases, diabetes, cardiac disease, and widely in the field of dentistry [56]. This therapy also has a long-standing history of being a potential source of dopaminergic cells that could be grafted into PD patients. Among the few areas affected in PD patients, caudate dopaminergic dysfunction and degeneration of dopaminergic neurons of SNpc are commonly seen in these patients, leading to Parkinsonian symptoms, such as cognitive impairment, depression, sleep, and gait problems [56]. It was observed that there was a baseline unilateral or bilateral caudate dopaminergic dysfunction in patients with early, untreated PD cohorts, specifically a significant reduction of caudate signal compared with controls at the time of clinical diagnosis. The reduction was associated with worse outcomes based on PD symptoms at the four-year follow-up. Studies have found the most promising stem cell types of embryonic stem cells (ESCs) and induced pluripotent stem cells (iPSCs) [57]. In a rat model of PD, there was efficacy in restoration of motor function, showing long-term survival and functionality, specifically how human ESC-derived DA neurons potentially regulate midbrain-to-forebrain projections and innervate target structures [58]. Using lesions to produce a dopaminergic-denervated striatum model of rats, the study grafted human ESC neurons to the striatum and found surviving transplants, which increased in volume, suggesting initial proliferation and potential maturation of transplanted cells. These neurons additionally provided extensive reinnervation throughout the caudate-putamen region and began the restoration of dopaminergic neurotransmission five months post-transplantation [58].

With the significant loss of dopaminergic neurons of the SN in PD pathology, there are currently no curative treatments, only treatments centered around symptom control focused on DA replacement therapy. Three main categories of PD medications include drugs that increase DA levels in the brain, drugs that affect other neurotransmitters in the body to ease PD symptoms, and ultimately medications that control non-motor symptoms of PD. Amongst the dopaminergic drugs, levodopa (L-DOPA), which results in general DA delivery with the ability to cross the BBB to areas of the brain other than the dopamine-deplete striatum, is administered with a peripheral dopa-decarboxylase inhibitor, carbidopa, to reduce peripheral side effects. Most of these drugs, while helping relieve motor symptoms, can have problematic side effects [59], such as L-DOPA, which can result in hallucinations and cognitive impairment due to off-target effects of the drug. In one case-study with a 65-year-old woman with an unclear diagnosis of PD, she was prescribed L-DOPA for motor symptoms, and while the patient had no symptoms of hallucinations or delusions, after years of increased doses of L-DOPA, the patient developed severe psychosis with impairment of social functioning [60]. It has also been shown that the post-commissural putamen of PD patients has increased permeability of the BBB using histologic markers of certain cell factors [61]. The study examined the integrity of the BBB through extravascular erythrocytes, which was found to be sevenfold higher in PD striatum compared to controls. The BBB dysfunction in the striatum of PD patients often results in L-DOPA-induced dyskinesia, reporting angiogenesis and increased permeability of the BBB in animal models of PD [62]. There is a need for a more targeted delivery of DA to the striatum, with DA being released in a physiological manner, minimizing off-target side effects, motor fluctuations, and dyskinesias seen with L-DOPA therapy. Additionally, BBB leakage is increased in PD [63]. The BBB permeability and cerebral blood flow are mainly controlled by endothelial cells, smooth muscles, and pericytes; thus, damage to the BBB has been associated with the accumulation of neurotoxins and hypoxia, leading to neuronal injury and loss [64]. This disruption contributes to neurodegeneration in the SNpc, and its connections have been seen in animal studies. Some dysfunction of the BBB transporter system was also noted in PD patients; however, further studies are needed. Studies have shown that subtle BBB disruption in PD has implicated the pathophysiology of areas such as the SN, white matter, and posterior cortical regions [64].

After several differentiation techniques and protocols, ESCs developed that closely resemble authentic nigral dopaminergic neurons, expressing genes such as LMX1A and FOXA2, important markers for the nigral dopaminergic phenotype [65]. Compared to ESCs, iPSCs are generated by reprogramming of an adult somatic cell into a stem cell through the expression of transcription factors that could induce pluripotency [66]. They could be differentiated into dopaminergic neurons using protocols similar to ESCs, which could serve as the basis of a useful cell-based treatment for PD. A potential benefit of iPSC over ESC grafts is that it would be possible to generate autologous grafts, using [67] the patient's own fibroblasts to produce a neural grafting product, negating the requirement for immunosuppression needed with ESC-derived grafts. Compared to ESCs, iPScs have extensive involvement in DNA repair. In many in-vivo studies, the dopaminergic cells in grafts could survive and make synaptic connections in the brains of rodents, resulting in motor and behavioral improvement. Once translated to clinical studies, there were logistical barriers and an inadequate supply of fetal tissue; however, there was also evidence that neural grafting could serve as a basis for a useful symptomatic treatment in PD. Stem cell therapy has great potential as a regenerative therapy; however, the questions have shifted to the delivery of these treatments rather than whether it is possible to generate a useful cell product [57].

Within PD patients, the dual syndrome hypothesis differentiates cognitively impaired PD patients into two groups. The first group includes the tremor-dominant patients with mild cognitive impairment, which is potentially due to dysfunction in the frontostriatal network, involving the caudate nucleus and prefrontal cortex, while the second group consists of patients with early postural instability and gait imbalance, expressing more visuospatial disturbances resulting from posterior cortical and temporal lobe dysfunction [68]. According to studies [68], caudate functional connectivity correlates with cognitive performance in PD by finding a strong positive correlation between the BC (betweenness centrality - a measure of networks involving nodes) of the right caudate nucleus and Montreal Cognitive Assessment (MoCA), which was used to select subjects for the study. Their data anticipated that the impairment stemming from the caudate nucleus, an important information hub, occurred due to the dopaminergic degeneration in the caudate nucleus in PD. Their findings of a positive correlation between the BC of the right caudate and MoCA scores support claims that the caudate nucleus is involved in PD and the associated cognitive deficits. With PET imaging, they also found that decreased dopaminergic function of the right caudate was related to slow processing time. Additionally, more prominent hypometabolism in the right caudate has been shown in cognitively impaired PD patients, supporting that caudate DA degeneration has been associated with cortical hypometabolism, which does not appear until after two years from initial diagnosis but is in slow progression over the later years. This further suggests that, while the prefrontal-caudate circuitry is intact in early-stage PD, the overall DA levels in the caudate nucleus are significantly lower than those of comparable normal subjects [69].

Discussion

KD and Stem Cell Therapy

This review aimed to understand the effects of a KD combined with stem cell therapy on PD patients' progression of disease, symptoms, and the pathophysiology behind PD. Certain dietary regimens have been associated with stem cell renewal and regeneration, specifically in the context of tumors and cancer. An HFD was found to induce a robust peroxisome proliferator-activated receptor delta (PPAR-δ) signature in intestinal stem and noon-ISC progenitor cells [70]. This diet enhances the self-renewal potential of intestinal organoid cultures in a PPAR-d dependent process, providing organoid-initiating capacity to progenitors. An HFD boosts ISC counts and crypt function, as observed in long-term HFD-fed mice. Researchers additionally observed that HFD-derived primary organoids generated more secondary organoids and had higher frequencies of ISCs subtypes compared to controls, suggesting that HFD potentially boosts intestinal stem cell regeneration.

As mentioned, current conventional treatments include drug regimens, such as L-DOPA and carbidopa for PD [71], particularly L-DOPA being the precursor to dopamine. These treatments effectively control motor symptoms of PD, targeting the degeneration of the SN in patients with PD. In contrast to dopamine, L-DOPA can cross the BBB and convert to DA in both the CNS and periphery. To enhance the effects of L-DOPA and decrease its side effects, carbidopa, a peripheral DA decarboxylase inhibitor, has been added as a combined therapy, allowing for more L-DOPA to cross the BBB. Its adverse effects, however, often range from nausea, headache, dizziness, to hallucinations, delusions, psychosis, and agitation in the elderly. Excessive daytime sleepiness can become a serious concern, and it is often recommended to discontinue the drug regimen if patients experience somnolence. L-DOPA has been linked to toxicity in various neuronal and non-neuronal cells, generating harmful free radical species and triggering apoptosis [72].

In efforts to alleviate and reduce these symptoms, KD serves to make a difference, combined with stem cell therapies, potentially moving beyond the management of PD and potentially eliminating the condition. While stem cell transplantation needs further development and consistent reproducible procedures, the diversity in the types of stem cells has shown tremendous promise. Transplanted ESCs could assist in the management of exogenous DA agents, relieving symptoms of L-DOPA-induced dyskinesia [73]. As previously mentioned, studies have demonstrated how transplantation of ESC into PD-induced rat models has resulted in notable improvements in motor dysfunction symptoms and symptoms of behavioral defects [74]. For instance, implanted ESCs primarily developed into neural grafts with mature ventral midbrain-like DA neurons, using molecular and enzyme markers. One study developed a novel therapeutic approach for PD, collecting fibroblasts from alpha-synuclein (SNCA) A53T transgenic mice, reprogramming them into iPS cells, knocking down expression of mutated SNCA in these iPS cells, and differentiating these cells into neural stem cells (NSCs). The NSC-shSNCA are ultimately transplanted into cortical areas of SNCA A53T transgenic mice. These mice were evaluated for the therapeutic effects of NSCs, observing that they had improved balance, coordination, and locomotion abilities following the transplantation [75]. In addition to improved PD-like symptoms in mice, mice transplanted with NSC-shSNCA cells exhibited extended lifespans, indicating that the transplantation could compensate for the loss of dopaminergic cells in these mice. As established, stem cell therapies have the potential to reprogram adult somatic cells into stem cells, generating iPSCs, using transcription factors, and this process could potentially be influenced by the body's conditions. By creating optimal conditions for the growth of stem cells, the reprogramming process can be more efficient and precise, increasing the number of dopaminergic neurons. For instance, metabolic changes and shifts can enhance the environment of the body to create optimal conditions for stem cell renewal and growth. Given the progressive success of stem cell transplants and grafts, KD can further support the therapeutic with its neuroprotective effects. A recent study showed that a longer-term medium-chain triglyceride KD (MCT-KD) significantly reduced MPTP-induced damage to dopaminergic neurons, induced antioxidant stress, and reversed oxidative stress in DA neurons [67]. The increased ketone bodies can inhibit neuroinflammation, alter gut microbiota, and consequently change the metabolism of SN neurons through gut microbiota metabolites. It was shown that the diet increased activities of enzymes and antioxidant concentrations, analyzing protein expression and phosphorylation of PI3K and Akt, key components of antioxidant stress in neurodegenerative diseases, including PD. Stem cell renewal and transplantation can be enhanced if implemented with KD as there would be fewer effects on other pathways and KD would simultaneously reduce the severity of PD symptoms. Mitochondrial damage and reactive oxygen species accumulation further lead to DA neuron loss; thus, KD has affirmed neuroprotection using ketone bodies as a substitute for energy. Substituting the main source of energy, glucose, with ketone bodies has many beneficial effects on the body, and studies have emphasized that the PI3K/Akt signaling pathway is crucial for the protection, which is often altered in PD models. Using KD to regulate oxidative stress levels by influencing molecular targets, patients with PD can potentially benefit from slower neurodegeneration and reduced effects of oxidative stress, such as less neuronal cell death and apoptosis. A combination of stem cell therapy and KD can regulate such pathways, which contribute to the development of PD, targeting one of the root problems in this disorder.

The growing field of the gut-brain axis also presents huge benefits for PD patients, utilizing both KD and stem cell therapy simultaneously. With diverse gut disorders, interventions, and dietary factors linked to PD development [76], dietary restrictions and KD can enhance PD therapeutics, particularly stem cell growth and differentiation in the SN. Different dietary paradigms converge on enforcing stem cell self-renewal across different tissue types without a clear known mechanism [77]. Certain factors have been identified to contribute to these convergent phenotypes, such as a decline in glucose availability and an increase in lipid utilization, stem cell-intrinsic glucose and fatty acid metabolism, and, finally, the degree of fasted/fed state pushes stem cells toward self-renewal or differentiation. For instance, stem cells of muscle, bone marrow, and hair follicle mainly remain in the relatively quiescent state of homeostasis, allowing them to preserve their self-renewing, tissue-regenerative capacity. Thus, caloric restriction enhances their preservation and function in both young and old animal models. While the specific mechanism behind this favorable state is unclear, it opens the opportunity to explore how NSCs respond to caloric restriction or even the KD [78]. Other studies have demonstrated that calorie restriction transiently enhanced proliferation of neural progenitor cells in young mice and prevented age-related loss of neurogenesis in the aged mice brains, specifically in the subventricular zone as seen with olfactory memory, microglial activation, and cytokine activity [79].

Limitations

This review has several limitations that must be considered when interpreting the conclusions. Firstly, much of the evidence supporting both KD interventions and stem cell-based therapies in PD has been derived from studies using in-vitro experiments and animal models. While these studies provide important insights, they do not address the complexity, heterogeneity, and chronic progression of PD in humans. Notably, there are currently no clinical trials evaluating the combined use of KD and stem cell therapy in PD. As such, any proposed synergistic benefit is hypothesis-generating rather than evidence-based. There are key limitations to stem-cell therapies. Substantial translational barriers exist, such as immune compatibility and long-term survival of transplanted cells, risk of tumorigenicity, variability of PD stage, and diversity within grafted neurons. Additionally, the adherence to a KD may be challenging for many patients, specifically older adults with comorbidities, and carries potential risks such as micronutrient deficiencies. Overall, rigorous clinical validation and carefully designed clinical trials are required before the combined therapy of KD and stem cell therapies can be considered disease-modifying strategies for PD. Table 1 presents the current therapeutics for PD [80,81].

**Table 1:** Current therapeutics for Parkinson's disease COMT - Catechol-O-methyltransferase; MAO - Monoamine oxidase Source: Refs [80,81]

Current and Future Therapies	Mechanism/Significance	Potential complications
Levodopa/Carbidopa combination	Levodopa or L-DOPA: Converts to dopamine in the brain. Carbidopa: Prevents levodopa’s breakdown before it reaches the brain [80,81].	Short half-life of levodopa causes fluctuations in the blood levels, causing a wearing-off phenomenon. Neurotoxicity such as hallucinations, delusions, and psychosis [80,81].
MAO inhibitors: Selegiline, Rasagiline, Safinamide	Increase the amount and duration of dopamine’s effects through inhibition of metabolism by MOA [81].	Variable reactions, lower rate of dyskinesia and motor complications but lacks neuroprotective effects clinically [81].
COMT inhibitors	Promotes levodopa entry into the brain via inhibition of levodopa metabolism in the periphery [81].	General adverse effects such as nausea, and vomiting, hallucinations, and dyskinesia [81].
Dopamine agonists	With longer half-lives than levodopa, deal with the wearing-off phenomenon in early treatment and the advanced stage [80,81].	General adverse effects of nausea, vomiting, orthostatic hypotension, and cardiac arrhythmia [80,81].
Alpha-synuclein targeting therapy	Includes immunization with anti-alpha-synuclein oligomer monoclonal antibodies and an inhibitor of misfolding of alpha-synuclein [81].	Currently investigated in clinical trials [81].
Anti-oxidative stress drugs	Includes iron chelators, myeloperoxidase inhibitors and analogs of antioxidant coenzyme Q10 [81].	Currently investigated in clinical trials [81].

## Conclusions

This review examined the role of stem cell therapy and KD in the context of PD therapy and outcomes. While conventional therapies have been effective, they also result in serious adverse effects for PD patients, specifically the elderly population. The fact that studies have found that PD has improved significantly with stem cell therapies in animal models and PD symptoms have also been managed effectively in these patients using a KD, we explored how a combination of the two could potentially alleviate the effects of current treatments. Further studies must be performed to develop efficient delivery systems of stem cell therapies for PD patients. Additionally, KD can produce many beneficial health effects, specifically for patients with neurodegenerative disorders; thus, implementation of this dietary regimen could potentially impact the course of PD. Additional studies with the mutual effects of combined stem cell therapy with KD must be performed to understand the effects of the combined therapy on PD patients. Long-term studies on PD patients would help build further understanding of the pathogenesis behind PD.
